# Unmet Health Needs of Roma Women in the Two Biggest Roma Communities in the Republic of Srpska, Bosnia and Herzegovina

**DOI:** 10.3389/fpubh.2020.00030

**Published:** 2020-03-10

**Authors:** Stela Stojisavljevic, Milkica Grabez, Kristefer Stojanovski

**Affiliations:** ^1^Department of Social Medicine, Public Health Institute of the Republic of Srpska, Banja Luka, Bosnia and Herzegovina; ^2^Faculty of Medicine, University of Banja Luka, Banja Luka, Bosnia and Herzegovina; ^3^Department of Health Behavior and Health Education, School of Public Health, University of Michigan, Ann Arbor, MI, United States

**Keywords:** Roma women, health insurance, health status, unmet health needs, barriers to health access

## Abstract

**Background:** Reasons for unmet health needs vary from individual to contextual determinants but are defined as the difference between needed health service and services actually received. Roma experience elevated health issues and challenging social conditions.

**Objective:** The aim of this study was to explore the unmet health needs and potential risk factors among Roma women living in the two biggest Roma communities in the Republic of Srpska.

**Method:** We conducted a health assessment of 183 adult Roma women in the Republic of Srpska. Unmet health needs were observed as the absence of needed medical supervision, despite having chronic conditions. We used logistic regression to assess the degree to which unmet health needs were related to the social determinants and the health status of Roma women.

**Results:** The majority of Roma women were married or were in an unofficial relationship (55.2%), were without schooling (62.8%), and were unemployed (88.5%). The results showed that 94.0% had health insurance, had a health card, and were registered with a family medicine doctor. Sixty percent reported having a chronic disease; however, 68.2% reported that their chronic disease was not medically supervised. Roma women that had less education, those who were unemployed, and those who were divorced or widowed women were more likely to have unmet health needs.

**Conclusion:** Roma women in Bijeljina and Prijedor have unmet health needs due to the circumstances they live in despite the fact that majority of them have health insurance and universal health access is legally guaranteed.

## Introduction

Roma persons are an ethnic group that live throughout Europe with a high concentration in the Balkans. The Roma population is the largest ethnic group in the Republic of Srpska (RS) of Bosnia and Herzegovina (BiH), although according to official statistical estimates, the Roma population has been decreasing in the last decade (1). Despite official statistical data, it is hard to estimate the total number of Roma population in the country. According to the official Population Census done in the RS in 2013, there are 1,974 Roma in the RS, while Roma's non-governmental organizations claim that there are between 3,000 and 4,000 Roma living in the RS (1, 2).

According to the Multiple Indicator Cluster Survey (MICS) conducted in 2012 in BiH, Roma are more socially excluded than the general population. This contributes to Roma in BiH living on the social margins and rarely able to participate in social, economic, and other activities (3). Majority of Roma in BiH and RS have law levels of education, which diminishes their ability to find employment and leads to poverty (3). Existing data show that Roma women in BiH have an even more difficult social position than Roma male counterparts. Roma women in BiH have less education, are more likely to be unemployed, are more financially dependent, are exposed to domestic violence, and are to be discriminated by their non-Roma counterparts (3). Although the number of studies on Roma health in BiH is limited, there is evidence that the Roma population have poorer health as compared to the national average.

Unmet health needs can be defined as the difference between health services that a person finds necessary for their health problem and services that they actually receive (4). Reasons for unmet health needs vary across individual and contextual determinants and between different population groups (5). Due to poor life conditions and other sociodemographic determinants, Roma people are more likely to have more unmet needs, and it could be an important indicator to measure access to health and health care. As some examples of the disparities, the morbidity and mortality rates are higher in Roma. Roma life expectancy is shorter, and infant mortality rates are higher in Roma (6). The Roma population often face frequent health problems, including a higher prevalence of tuberculosis, cardiovascular diseases, and other communicable and non-communicable diseases (7, 8).

Universal health care access is legally guaranteed to all the citizens of the RS, BiH (9). Despite the fact that the health insurance system in the RS is based on mandatory health insurance for all citizens, there are persons who are excluded, such as those who had never been employed, of which are the majority of the Roma population in the country (10). The range of services provided by the national health insurance scheme is limited, and often out-of-pocket payments occur for medical checkups, medicines, and medical devices. Given that Roma live in abject poverty, such payments are often not feasible, particularly for Roma women (11). The aim of this study was to explore the unmet health needs and potential risk factors among Roma women living in the two biggest Roma communities in the RS.

## Method

### Study Design and Selection of Participants

Between August and December 2018, the Roma health improvement program was implemented in the two biggest Roma communities, settled in the towns of Prijedor and Bijeljina in the RS. The health improvement program's goal was to provide medical care. Therefore, the health assessment was related to the identification of Roma people with health problems and not under medical supervision. We also chose the not under medical supervision outcome to explore gaps in the health system in RS despite the fact that Roma are guaranteed health insurance. The program had three components: (1) assessment of the health status of the Roma population; (2) provision of information on health insurance and health entitlement; and (3) education of the Roma population. The Public Health Institute of the RS implemented the program in close cooperation with Roma non-governmental associations (NGOs) and Roma health mediators. The Roma health mediators are usually engaged by public institutions with the goals of (1) improving the communication between Roma communities and public institutions, (2) increasing health literacy of Roma, and (3) facilitating between Roma communities and health institutions to help improve health and social integration. Since the health mediators are from the Roma communities, they are more familiar with the challenges faced by the communities. Health mediators are trained to address different Roma needs, mainly related to health, as well as education and getting necessary documents and identification (12). We used a time location sampling (13) to recruit Roma participants. The eligibility criteria included declaring oneself as of Roma nationality, being at least 18 years old, and providing written informed consent to the interviewer. All adult Roma people who were present at the homes at the time of the health assessment were interviewed. If Roma families were not present at the first visit, they would be visited a second and third time. The health assessment was conducted house to house, with voluntary participation of the Roma population. Out of 196 Roma families, 172 families (87.7%) were present and participated in the interview. In total, 183 Roma women were assessed and interviewed: 82 from Prijedor and 101 from Bijeljina.

### Data Collection

The Roma health mediators conducted the health assessment part of the program and conducted interviews with Roma. The survey part of the health assessment program contained close-ended questions on sociodemographic status, socioeconomic status, health status, such as chronic diseases, and health system access, such as health insurance status, health system registration, and medical supervision. All interviews were administered in the Serbian language, one of the three official languages in the RS. The Roma health mediators were inhabitants of the Roma's communities, were supported by the Roma NGOs active in the settlements, were native speakers, and had secondary education. The health assessment was conducted house to house, with voluntary participation of the Roma population. The information of the assessment and its purpose were provided to all Roma who settled in Prijedor and Bijeljina and participation was offered.

Prior to health assessment, Roma mediators were trained by the team at the Public Health Institute in data collection and protocols. We developed a questionnaire, which was pretested, in close coordination with the Roma representatives.

If Roma families were not present at the first visit, then they would be visited a second and third time. Out of 196 Roma families, 172 families (87.7%) were present and participated in the interview. The remaining 24 families were not found after three visits. Among the 172 families, there were a total of 183 Roma women who met the eligibility criteria and were interviewed, 82 from Prijedor and 101 from Bijeljina. Roma participants could decide to remain anonymous or provide personal data depending on their comfort. The data when entered into the database were de-identified. The Roma who accepted participation in the health assessment/interview provided written consent to the health mediator.

### Data Analysis

We conducted statistical analysis using IBM SPSS Statistics (version 20.0). Baseline demographic characteristics, such as age, education status, employment status, and marriage status were summarized using frequencies and percentages for categorical characteristics, and mean and standard deviations for continuous variables. Age was categorized by guidelines on standard international classification using single years at the level of age 18–24, and 10 years groups for population 25+ (14). We categorized education into four categories: without school, primary school, secondary school, and tertiary education (faculty and above). Marriage status was categorized as married, unofficial relationship, divorced, widowed, and single. Employment was categorized as permanently employed, temporarily employed, and unemployed. Symmetric 95% confidence intervals (95% CI) were calculated for frequency tests. The categorical variables were compared using chi-square (*X*^2^) measures of association where appropriate. All *p*-values <0.05 using a two-tailed test were considered to be significant. The outcome variable was health status, which was observed as having any of the chronic diseases diagnosed in the health assessment (yes, any vs. none). The secondary outcome of unmet health needs was categorized as not being under medical supervision. Logistic regression was conducted to evaluate the impact of sociodemographic characteristics on the likelihood of having a diagnosed chronic disease and unmet health needs.

## Results

The sample included 183 Roma women with an average age of 40.9 years (*SD* = 16.1). A significant proportion of Roma women were without schooling 62.8% (95%CI = 51.9–75.4), and half live with a partner, 55.2% (95% CI = 45.0–67.1), whether married or unofficial relationship. The majority of Roma women were unemployed, 88.5% (95%CI = 75.42–100.00), while only 2.7% (95%CI = 0.80–6.00) were permanently employed (Table 1).

**Table 1:** Sociodemographic characteristic of the sample of Bosnian Roma women (*n* = 183).

	**Number**	**Percent**	**95% CI**
Age group (year)			
18–25	35	19.1	13.3–26.6
26–35	42	22.9	16.5–31.0
36–45	39	21.3	15.2–29.1
46–55	30	16.4	11.1–23.4
56–65	20	10.9	6.7–16.9
>65	17	9.3	5.4–14.8
Education level			
Without school	115	62.8	51.9–75.4
Primary school	49	26.8	19.8–35.4
Secondary school	19	10.4	6.2–16.2
Marital status			
Single	27	14.8	9.7–21.5
Married	80	43.7	34.7–54.4
Unofficial relationship	21	11.5	7.1–17.5
Divorced	20	10.9	6.7–16.9
Widowed	35	19.1	13.3–26.6
Employment status			
Unemployed	162	88.5	75.42–100.0
Permanent employment	5	2.7	0.8–6.00
Temporary employment	8	4.4	1.9–8.6
Other	8	4.4	1.9–8.6

*95% CI, 95% confidence interval*.

The results showed that 94.0% of Roma women stated that they had health insurance and a health card and that they were registered with family medicine doctors. However, few women, 74.9%, were registered with a gynecologist (Table 2). Older women more often did not have health insurance, as compared to their younger counterparts (*p* = 0.026).

**Table 2:** Health insurance status and registration with health authorities.

	**Number**	**Percent**	**95% CI**
Health insurance			
No	11	6.0	3.1–19.7
Yes	172	94.0	80.5–100.0
Health card			
No	11	6.0	3.0–19.7
Yes	172	94.0	80.5–100.0
Registration at family doctor			
No	11	6.0	3.0–19.7
Yes	172	94.0	80.5–100.0
Registration at gynecologist			
No	46	25.1	18.4–33.5
Yes	137	74.9	62.9–88.5
Reported chronic disease			
No	73	39.9	31.3–50.2
Yes	110	60.1	49.4–72.4

*FM, family medicine; GYN, gynecologist; 95% CI, 95% confidence interval*.

Sixty percent (*n* = 110) of Roma women had at least one chronic disease diagnosed: of these, 91.8% had health insurance. There were significantly more women with diagnosed chronic diseases who were not under medical supervision (68.2%, *p* < 0.001) in comparison to those under medical supervision (31.8%) (Table 3). The main reasons for not being under medical supervisions were 33.6% (*n* = 37) lacked money for medical checkups and 34.5% lacked money for prescriptions (*n* = 38).

**Table 3:** Relationship between health status, health insurance, and medical supervision.

	**Health Status**		
	**None *n* (%)**	**Chronic disease *n* (%)**	***p***
Health insurance			
Yes	71 (97.3)	101 (91.8)	
No	2 (2.7)	9 (8.2)	0.113
Total	73 (39.9)	110 (60.1)	
Unmet health needs			
Not under medical supervision	0 (0)	75 (68.2)	
Under medical supervision	73 (100.0)	35 (31.8)	<0.001
Total	73 (39.9)	110 (60.1)	

As shown in Table 4, the adjusted odds of not being under medical supervision were 1.9 times higher (95% CI = 0.7–5.6) among divorced Roma women and 1.6 times higher (95% CI = 1.0–4.5) among widowed women, as compared to married women. Unemployed women were 2.3 times more likely to not be under medical supervision (AOR = 2.3, 95% CI = 0.7–7.9) as compared with employed women. Further, Roma women without any education (AOR = 1.6, 95% CI = 0.4–7.2) had higher odds for unmet health needs, as compared to Roma women with secondary education. Roma women, after adjusting for other factors, who had a diagnosed chronic disease had higher odds of not being under medical supervision, as compared to those with no chronic diseases (AOR = 53.3, 95% CI = 11.7–243.2).

**Table 4:** Odds ratios and associated 95% confidence intervals for adjusted multiple logistic regression of sociodemographic and health status associated with met health needs.

**Predictor**	**OR**	**95% CI**		***p***
		**Lower**	**Upper**	
Intercept	4.4	1.4	13.7	0.012
Age	1.1	1.0	1.06	0.006
Marital status^a^				
Divorced	1.9	0.7	5.6	0.214
Widowed	1.6	1.0	4.5	0.447
Single	0.4	0.1	1.4	0.142
Unmarried relationship	0.2	0.0	0.8	0.026
Educational status^b^				
Without primary school	3.3	0.8	13.5	0.111
Primary school	1.6	0.4	7.2	0.548
Employment^c^				
Unemployed	2.3	0.7	7.9	0.181
Health issue				
Chronic disease diagnosed	53.3	11.7	243.2	<0.001

a *Marital status, reference group is married*.

b *Education, reference group is secondary school*.

c *Employment, reference group is employed*.

## Discussion

Our results indicate that more than half of the interviewed Roma women in the RS, BiH had at least one chronic disease diagnosed. However, disparities existed such that Roma women without any education, who were unemployed, and who were divorced or widowed despite having health insurance were not under medical supervision. Roma women in the RS have high unmet health needs.

The Constitution of the RS guarantees equal right to health care to all citizens regardless of sex or race, or national or social origin (15). There are also important legal documents that regulate health care: the Law on Health Care, the Law on Health Insurance, and the Law on Social Protection (9, 16, 17). According to these regulations, all citizens in the RS have equal rights to health care regardless of national, religious, or other affiliations. Our results showed that some of these laws might be working because majority of Roma women had health insurance.

Our study aligns with findings from North Macedonia where Roma frequently lack access to health care, although the rights to health are stipulated in the Macedonian Constitution (18). Other Central and Eastern European countries had varying rates of health insurance coverage among Roma. For example, in Slovakia, only 2.8% of Roma were without health insurance, while 59.7% were without health insurance in Moldova and 67.7% in Albania (19).

Despite the compulsory health insurance system in the RS and the constitutionally guaranteed rights to health, there are other social determinants, such as poverty and education that override Roma women's capacity to address their health needs. Ekmekçi found a link between financial constraints and poorer socioeconomic determinants that leads to a high level of unmet health needs (20). Economic barriers include formal and informal payment of charges for health services, lack of financial resources for transport to the health care institutions, inability to pay communication costs, and limited funds for prescriptions or to pay health insurance costs (21).

For Roma women, the lack of money to cover medical costs or prescriptions is a huge barrier in gaining access to health care. As noted, 60% of Roma women who participated in our study reported one or more chronic diseases diagnosed, but more than two-thirds of them reported lack of health care and unmet health needs. The literature describes an evident association between social determinants and health status such that poor social status, unemployment, lack of occupational safety, and illiteracy all have a negative impact on health (22–24). Although there are various reasons for unmet health needs, such as distance, waiting list, and limited human resources, numerous studies confirmed that financial constraints are one of the main reasons for unmet health needs (25). Unmet health needs often contribute to poorer health, especially in the most vulnerable population, such as the Roma population. Unmet health needs of Roma women in the RS of BiH are higher than in European countries. According to the Organization for Economic Co-operation and Development (OECD) report, the proportion of population who reported unmet health needs vary from 1.3% in Denmark to 16% in Sweden, but the main reason for not having medical checkups and treatment is the lack of finances (26). Data from Greece and Estonia confirm that about 10% of the general population have unmet health needs, and most of them fall in the low-income population group (26). Such global findings indicate that those who are most vulnerable, such as Roma and other poor socioeconomic groups, suffer the most from unmet health needs. As in many other studies, our findings point the importance of the relationship between social determinants of health and health status and confirm its evident association (27–29). Roma women with lower education status, who were unemployed, and who were living out of a partnership showed a higher likelihood of experiencing unmet health needs. The results of the study conducted in the RS, BiH support the findings from other studies conducted in Southeast Europe reporting unmet health needs in the Roma population as well as the challenge of health insurance coverage (24, 30). Sociodemographic characteristics of Roma women in the RS are one of the many contributors to unmet health needs and being without medical supervision.

### Study Limitation

The authors consider several limitations of this work. The study was cross-sectional, which hinders the ability to make casual inference, and the reverse associations could be true (e.g., not being under medical supervision is a reason for why many have a chronic disease). However, we did explore reasons for lacking medical supervision, which were predominantly related to finances, which supports global literature on the topic. The second is that we did not compare Roma women health status with their non-Roma neighbors so it was not possible to observe disparities in health insurance coverage and unmet health needs; however, many other studies have explored these disparities (31). The study did not provide the possibility of a detailed assessment of the barriers to health care access and the reasons behind barriers to obtaining health care. As the study was implemented as a part of the health improvement program, the Ethical Request was not obtained, although the study was implemented based on the principles of Helsinki Declaration and all participants provided written consent. The health improvement program also limited our ability to select the desired indicators given that the program was focused on ensuring the medical supervision of chronic diseases. Despite the limitations, the findings are aligned with the research among the Roma in other counties, and the study calls for a more detailed research, exploring the health status of Roma women and its social economic determinants.

### Conclusion

Roma women in the two biggest Roma communities in the RS face barriers to health care access and have unmet health needs despite a high level of health insurance coverage, which is guaranteed by the RS Constitution and other legal documents. Legal measures of access to health care do not necessary provide equal opportunities to Roma women to exercise their right to health and meet their health needs. There is a need for a detailed assessment of the health status of Roma women in the RS and barriers to health care and to assess changes to health over time.

## Data Availability Statement

The datasets collected for this study will not be made available for the public. We cannot provide these data due to two reasons: (1) potential privacy infringement and (2) ethical consideration for the study was not obtained prior to data collection.

## Ethics Statement

Ethical review and approval was not required for the study on human participants in accordance with the local legislation and institutional requirements. The patients/participants provided their written informed consent to participate in this study.

## Author Contributions

SS was involved in the design of the questionnaire for health assessment, data input into IBM SPSS, data interpretation, and draft of the manuscript. MG and KS conducted the data analysis and contributed to the data interpretation and manuscript draft.

### Conflict of Interest

The authors declare that the research was conducted in the absence of any commercial or financial relationships that could be construed as a potential conflict of interest.
